# Prognosis of the Keratinizing Squamous Cell Carcinoma of the Tongue Based on Surveillance, Epidemiology, and End Results Database

**DOI:** 10.1155/2023/3016994

**Published:** 2023-02-24

**Authors:** Hai Yu, Shuping Xie, Xinkai Zheng, Qiqi Zhao, Xichun Xia, Wai-Kit Ming, Leong Nga Cheng, Xi Duan, WenHsien Ethan Huang, Fang Huang, Jun Lyu, Liehua Deng

**Affiliations:** ^1^Department of Dermatology, The First Affiliated Hospital of Jinan University, Jinan University Institute of Dermatology, Guangzhou, China; ^2^School of Basic Medicine and Public Health, Jinan University, Guangzhou, China; ^3^Department of Dermatology, The Fifth Affiliated Hospital of Jinan University, Heyuan, China; ^4^Institute of Biomedical Transformation, Jinan University, Guangzhou, China; ^5^Department of Dermatology, Zhuhai People's Hospital (Zhuhai Hospital Affiliated with Jinan University), Jinan University, Zhuhai, China; ^6^Department of Infectious Diseases and Public Health, Jockey Club College of Veterinary Medicine and Life Sciences, City University of Hong Kong, Hong Kong, Hong Kong SAR, China; ^7^Department of Dermatology, Kiang Wu Hospital, Macau, Macau SAR, China; ^8^Department of Dermatology, Affiliated Hospital of North Sichuan Medical College, Nanchong, China; ^9^Gene Hope Clinic, Taipei, China; ^10^Department of Clinical Research, The First Affiliated Hospital of Jinan University, Guangzhou, China

## Abstract

**Background:**

The objective of this study is to determine the prognostic factors of keratinizing squamous cell carcinoma of the tongue (KTSCC) and to establish a prognostic nomogram of KTSCC to assist clinical diagnosis and treatment.

**Methods:**

This study identified 3874 patients with KTSCC from the Surveillance, Epidemiology, and End Results (SEER) database, and these patients were randomly divided into the training (70%, (*n* = 2711) and validation (30%, *n* = 1163) cohorts. Cox regression was then used to filter variables. Nomograms were then constructed based on meaningful variables. Finally, the concordance index (C-index), net reclassification index (NRI), integrated discrimination improvement (IDI), calibration charts, and decision-curve analysis (DCA), were used to evaluate the discrimination, accuracy and effectiveness of the model.

**Results:**

A nomogram model was established for predicting the 3-, 5-, and 8-year overall survival (OS) probabilities of patients with KTSCC. The model indicated that age, radiotherapy sequence, SEER stage, marital status, tumor size, American Joint Committee on Cancer (AJCC) stage, radiotherapy status, race, lymph node dissection status, and sex were factors influencing the OS of patients with KTSCC. Verified by C-index, NRI, IDI, calibration curve, and DCA curve, our model has better discrimination, calibration, accuracy and net benefit compared to the AJCC system.

**Conclusions:**

This study identified the factors that affect the survival of KTSCC patients and established a prognostic nomogram that can help clinicians predict the 3-, 5-, and 8-year survival rates of KTSCC patients.

## 1. Introduction

Tongue cancer refers to tumors occurring in the back of the tongue, two-thirds of the front of the tongue, the side edge of the tongue, and the bottom of the tongue. It is believed that the incidence of tongue cancer rises with age. However, there has been a noticeable rise among patients under 45, particularly among women, in recent years [1]. More than 90% of tongue cancers are tongue squamous cell carcinomas (TSCC), and their mortality rate has increased dramatically in recent years. Human papillomavirus, alcohol, and tobacco are indicated as risk factors for TSCC [2–4]. The WHO divides oral tongue squamous cell carcinoma into four classes based on the level of keratinization and differentiation of cancer cells. Grade I is well differentiated and has >50% keratinization (K4), grade II is moderately differentiated and has 20–50% keratinization (K3), grade III is poorly differentiated and has 5–20% keratinization (K2), while grade IV is undifferentiated and has 0–5% keratinization (K0-K1) [5]. Keratinizing squamous cell carcinoma of the tongue (KTSCC) is classified as having the keratinizing subtype if any amount of keratinization is present, which accounts for approximately 50% of TSCC [6]. The prognosis of different histological types of TSCC is significantly different. The 5-year survival rate of KTSCC is >80%, while the 5-year survival rate of hypokeratinizing and nonkeratinizing squamous cell carcinoma is >50% [1, 7]. Though KTSCC has a relatively good prognosis and low malignancy, the tongue has abundant blood vessels and lymph nodes, and the primary tumor is prone to metastasizing. Because of this, Cooper et al. found that KTSCC had both lower disease-free and disease-specific survival rates compared with other nonkeratinizing oral squamous cell carcinoma, indicating that keratinization is an important factor that affects the prognosis [8]. However, previous research into clinical prognosis assessments of patients with KTSCC have been inadequate, and hence a more accurate clinical prognosis assessment scheme is still needed to improve the reliability of predictions by doctors and improve the survival rate of patients.

The current standard method for evaluating the prognosis of various tumors is the unified tumor staging system developed by the American Joint Committee on Cancer (AJCC) [9, 10]. However, this method is only based on the histological characteristics of tumors and ignores human sociology and individual characteristics, such as sex, race, living environment, and other potentially important factors, which often results in highly inaccurate predictions [11]. Prognostic nomograms not only incorporate oncological characteristics but also combine the demographic and sociological characteristics of patients to more reasonably and accurately estimate their prognoses, making them more valuable for clinical applications [12]. Therefore, our study established a KTSCC prognostic nomogram using data from KTSCC patients in the Surveillance, Epidemiology, and End Results (SEER) database. The nomogram can be used to guide clinicians in evaluating the prognosis of KTSCC patients.

## 2. Methods

### 2.1. Data Source

Information on patients with KTSCC was obtained from the SEER subdatabase (SEER Research Plus Data, 17 Registries, Nov 2021 Sub (2000–2019)) using SEER*∗*Stat software (version 8.4.0.1) [13, 14]. The examined variables included the third edition of the International Classification of Cancer Diseases (ICD‐O‐3), age, tumor size, AJCC stage, race, SEER summary stage, sex, surgery status, marital status, radiotherapy status, lymph node dissection status, radiotherapy sequence, chemotherapy status, income, and vital status. Diagnoses made during 2004–2015 were selected. All relevant information was obtained from the SEER database (https://seer.cancer.gov/), and consent was not required from the patients [15, 16]. All-cause mortality in KTSCC was the outcome of this study.

### 2.2. Data Selection Criteria

**Figure 1:**
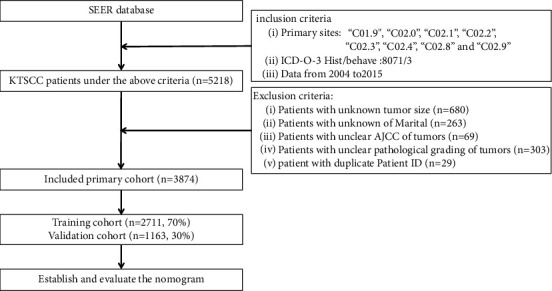
Research flowchart.

The following methods were used to extract KTSCC patients from the SEER database: The inclusion criteria: primary sites codes: “C01.9,” “C02.0,” “C02.1,” “C02.2,” “C02.3,” “C02.4,” “C02.8,” and “C02.9;” ICD-0-3 Hist/behave codes: 8071/3. Data from 2004 to 2015. A total of 5218 KTSCC patients were screened out using the above inclusion criteria. However, 680 patients were excluded because of unknown tumor size, 372 due to unclear AJCC stage, 263 due to unknown marital status, and 29 due to duplicate patient IDs. Finally, 3874 patients with KTSCC were included, who were randomly divided into the training (70%, *n* = 2711) and validation (30%, *n* = 1163) cohorts (Figure 1).

### 2.3. Statistical Analysis

Categorical variables were expressed as percentages, and differences among the variables were evaluated. Continuous variables were expressed using median and interquartile values. A prognostic nomogram for KTSCC was then established according to the variables screened using Cox regression. The concordance index (C-index) and time-dependent area under the receiver operating characteristic curve (AUC) were used to test the discrimination performance between the predicted value from this model and the actual value. Quantitative evaluation indicators such as net reclassification improvement (NRI) and integrated discrimination improvement (IDI) index were also used to evaluate whether the predictive ability of the model was improved over that of previous models in a more comprehensive and multilevel manner [17, 18]. The accuracy of the nomogram was tested based on the fit between the calibration and standard curves [19]. The validity of the nomogram was assessed using decision-curve analysis (DCA) [20].

SPSS Statistics software (version 27.0, Chicago, IL, USA) and R software (version 4.0.1; https://www.Rproject.org) were used for the statistical analysis, and statistical results were considered significant at *p* < 0.05.

## 3. Results

### 3.1. Basic Patient Information

Both cohorts had a median age of 61 years, and they were predominantly male (62.6% and 64.2% in the training and validation cohorts, respectively), white (84.6% and 86.2%), and married (56.6% and 58.9%). Tumor size is mainly less than 2 cm (40.6% and 40.5%), followed by 2–4 cm (32.2% and 30.5%). AJCC tumor stages were evenly distributed. Most of the tumors were localized and regional, and few patients had distant metastasis. More patients in both cohorts received surgery (79.6% and 78.4%), fewer received chemotherapy (31.5% and 31.9%), about half of the patients received radiotherapy, and patients who received radiotherapy were more likely to receive it after surgery (31.5% and 31.9%) in both cohorts. Most patients opted for postoperative lymph node dissection (57.5% and 55.8%), and most had middle incomes in both cohorts (51.2% and 49.3%). The median survival times Table 1 in the training and validation cohorts were 53 and 56 months, respectively, with no significant difference.

### 3.2. Variable Selection

Age, AJCC stage, sex, SEER tumor stage, marital status, tumor size, surgery status, radiotherapy status, race, chemotherapy status, lymph node dissection status, radiotherapy sequence, and income were analyzed using multivariate Cox regression. The final screening results were as follows: age (hazard ratio (HR) = 1.032, *p* < 0.001), female (vs. male: HR = 0.886, *p* < 0.05), black (vs. white: HR = 1.238, *p* < 0.05), single (vs. married: HR = 1.553, *p* < 0.001), other marital status (vs. married: HR = 1.353, *p* < 0.001), size = 2–4 cm (vs. ≤2 cm: HR = 1.346, *p* < 0.01), size >4 cm (vs. ≤2 cm: HR = 1.768, *p* < 0.001), AJCC stage II (vs. stage I: HR = 1.348, *p* < 0.05), AJCC stage III (vs. stage I: HR = 1.379, *p* < 0.05), AJCC stage IV (vs. stage I: HR = 1.586, *p* < 0.001), regional metastasis (vs. localized: HR = 1.342, *p* < 0.01), distant metastasis (vs. localized: HR = 1.951, *p* < 0.001), no radiotherapy (vs. radiotherapy: HR = 1.543, *p* < 0.001), radiotherapy before and after surgery (vs. no radiotherapy: HR = 2.429, *p* < 0.001), and no/unknown regional lymph node dissection (vs. regional lymph node dissection: HR = 1.196, *p* < 0.05) (Table 2).

### 3.3. Nomogram Creation and Evaluation

**Figure 2:**
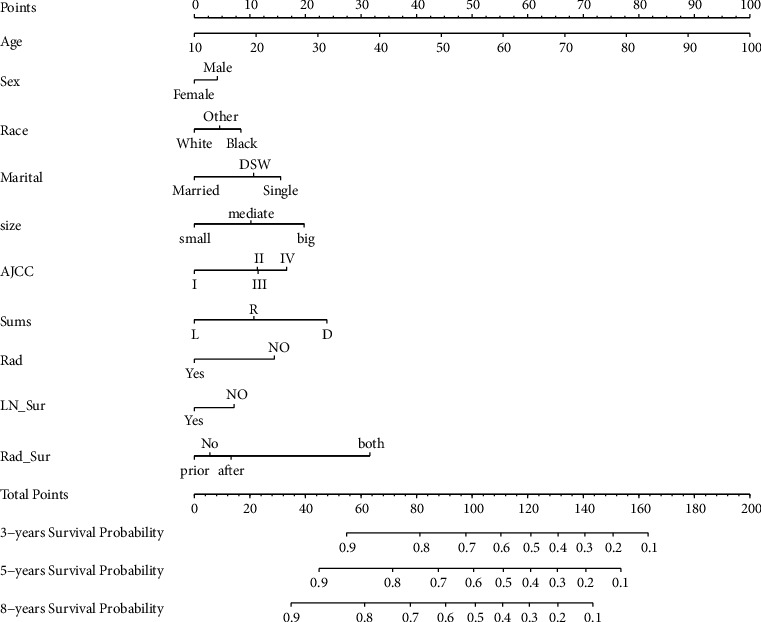
Nomogram predicting 3-, 5-, and 8-year OS probability. Sums: SEER summary stage; rad: radiotherapy status; chemo: chemotherapy status; LN_sur: regional lymph node dissection after surgery; rad_sur: sequence of radiation.

We used the final results from the multivariate Cox regression to construct a nomogram (Figure 2) for predicting the probabilities of overall survival (OS) at 3, 5, and 8 years in patients with KTSCC. The results of the nomogram indicated that age has the greatest impact on KTSCC prognosis, followed by radiotherapy sequence, SEER stage, tumor size, AJCC stage, marital status, radiotherapy status, race, lymph node dissection status, and finally, sex.

**Figure 3:**
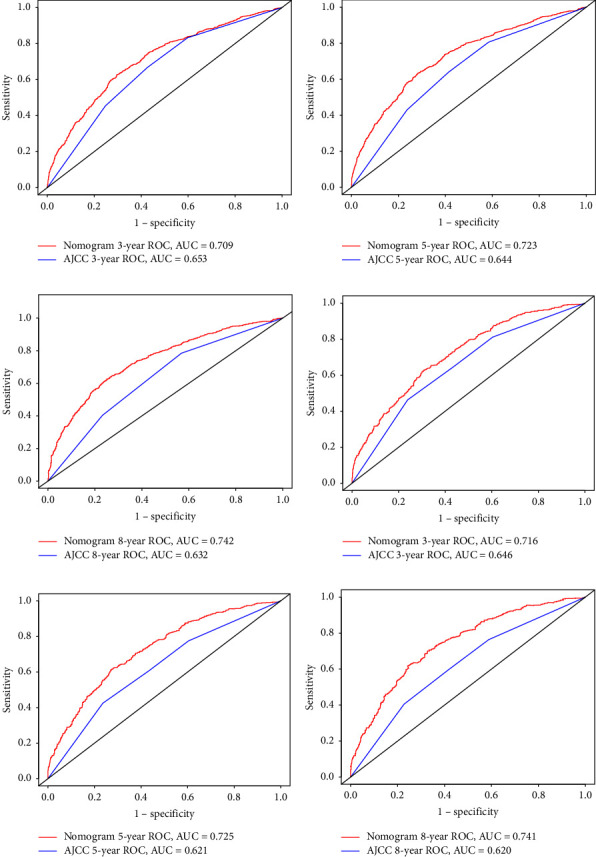
ROC curves. The area under the ROC curve (AUC) of the new nomogram and AJCC. Both training cohort (a–c) and validation cohort (d–f) show that the new nomogram predictive ability is better than that of AJCC.

The next step was to evaluate the nomogram. The C-index in both the training (0.688) and validation (0.691) cohorts was better than that of the AJCC staging system (0.617 and 0.611). The AUC values for the 3-, 5-, and 8-year OS probabilities were 0.709, 0.723, and 0.742 in the training cohort and 0.716, 0.725, and 0.741 in the validation cohorts; the corresponding values for the AJCC staging system were 0.653, 0.644, 0.632 and 0.646, 0.621, 0.620, respectively. These findings indicated that the new model had better predictive power than the AJCC staging system (Figure 3).

**Figure 4:**
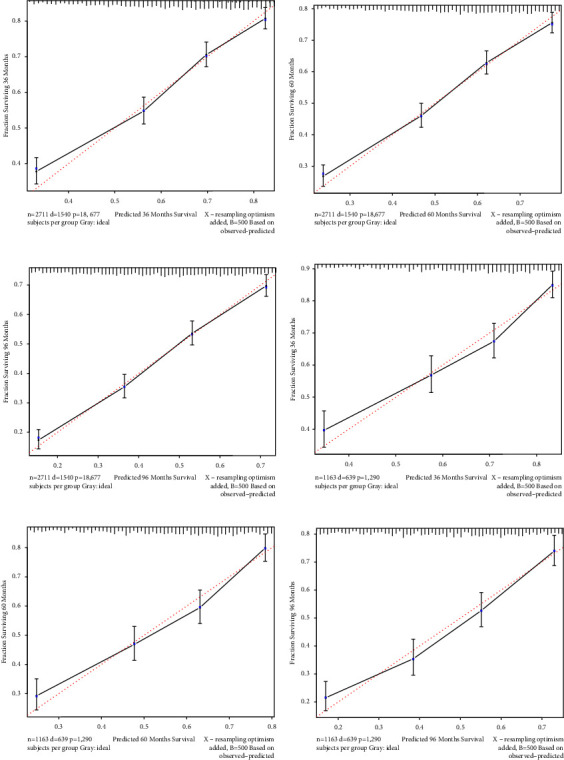
Calibration curves for the new nomogram. Both the *t* training cohort (a–c) and validation cohort (d–f) show that the calibration curves of the new nomogram are very close to the standard curve, and the points on the curve are evenly distributed.

The NRI values for evaluating OS probability in the training cohort were 39.6% (95% CI = 34.04–49.16%) at 3 years, 47.0% (95% CI = 41.59–55.63%) at 5 years, and 56.3% (95% CI = 50.42–68.21%) at 8 years, and for the validation cohort were 45.17% (95% CI = 29.40–58.72%), 53.30% (95% CI = 40.93–63.84%), and 60.18% (95% CI = 48.54–72.97%), respectively. The IDI values for OS in the training and validation cohorts were 10.5% and 10.6%, respectively, at 3 years (*p* < 0.001), 11.2% and 11.6% at 5 years (*p* < 0.001), and 11.5% and 11.9% at 8 years (*p* < 0.001). The NRI and IDI values were both higher than zero, indicating that the predictive power of the new model is better. The calibration curve indicated that the overall slopes of the curves of the training and validation cohorts were very similar to the reference line, and the points on the line were evenly distributed, indicating that our model had the strong predictive ability (Figure 4).

**Figure 5:**
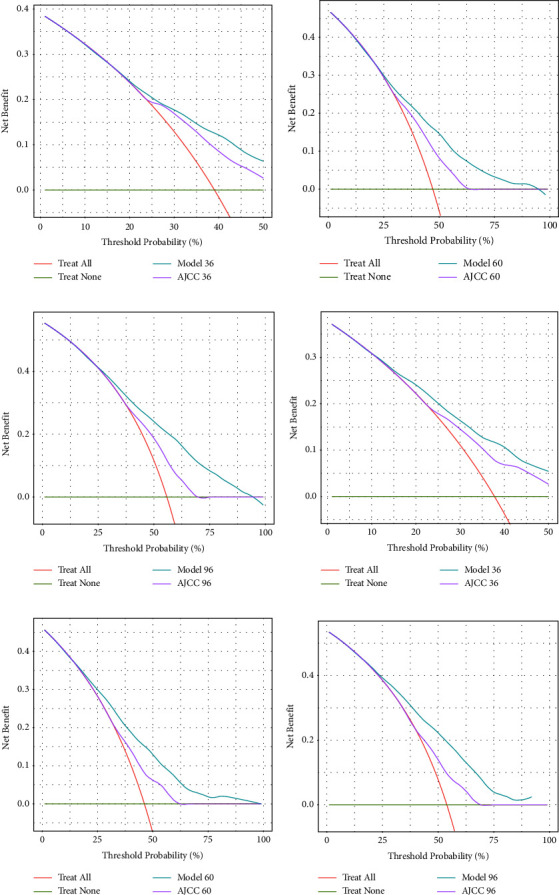
Decision curve analysis curves. Decision curve analysis of the training cohort (a–c) and validation cohort (d–f) for 3-, 5-, and 8-year cancer overall survival probability.

Figure 5 shows the results from the DCA of the nomogram for patients with KTSCC. It indicates that the net benefit of this model in predicting survival probability was greater than that of the AJCC system, indicating that the nomogram that we have established is effective.

## 4. Discussion

KTSCC is a subhistological type of squamous cell carcinoma of the tongue. To the best of our knowledge, TSCC is the most researched at present, and few people study KTSCC independently and systematically [21, 22]. The incidence and mortality of KTSCC are increasing, and the age of onset is getting younger. It is important for the prognosis study of KTSCC.

The AJCC system is currently available to assess the prognoses of most tumors but this method is based only on tumor histology, and does not take into account sociological and individual factors such as the economic status, race, and treatment conditions of patients. Our new nomogram model incorporated various comprehensive factors, including economic status, race, marital status, treatments, and other factors that independently affect the prognosis of KTSCC, and it can better predict the OS rate of patients with KTSCC. Comparing it with the AJCC staging system revealed that our new model can provide more accurate information, and for clinicians to use when performing diagnoses and formulating treatment plans for patients.

Age has been an independent factor affecting TSCC prognosis. Best et al. [23] found that 56.1% of young patients with TSCC had lymph node metastasis. However, the prognosis deteriorated as age increased, which may be related to excessive drinking and smoking and other factors [24]. The incidence and recurrence of TSCC in young patients have also recently been increasing [4]. Age was also the strongest risk factor for KTSCC prognosis in our new nomogram. For sex and race, like most TSCC [25], females with KTSCC have better OS than males, and black patients have significantly worse prognoses than white patients. We also found that single and divorced individuals had worse outcomes than married individuals. Studies have found that unmarried patients are more likely to have metastasis in various cancers, and the risk of death due to cancer also increases significantly, which may be related to poor compliance as well as the relatively low economic and educational levels of unmarried patients [26, 27]. Studies have found that the early diagnosis rate of most cancers is higher for married patients than for unmarried patients [28]. We, therefore, plan to conduct future prospective experiments to confirm the exact relationship between radiotherapy status and KTSCC prognosis.

This study also found that among the factors influencing KTSCC prognosis, tumor size, AJCC stage, SEER tumor stage, radiotherapy status, and postoperative lymph node dissection also affected the overall survival probability. Our findings indicated that larger tumors caused worse KTSCC prognoses. Similarly, in SEER staging, more-widespread tumor metastasis is associated with a worse prognosis, with distant metastasis having the worst prognosis. These results were closely related to the special location of the tongue that is rich in blood vessels with its adjacent lymphatic vessels. With the development of tumors, localized invasion, and lymph node metastases often occur at an early stage, and distant metastasis eventually occurs. Tumor metastasis will adversely affect the prognosis.

It is worth noting that our study found that radiotherapy status and postoperative lymph node dissection were protective factors for KTSCC prognosis. The current main treatment method for KTSCC is surgery. Postoperative selective neck lymph node dissection can increase the OS of patients and control recurrence. Consistent with the study by Kurita et al. [29] on oral squamous cell carcinoma, this study found that postoperative lymph node dissection was beneficial to the clinical prognosis of patients. However, surgical treatment can affect the speaking and eating functions of patients, while radiotherapy and chemotherapy can better protect their pronunciation, eating, and other functions and improve their quality of life [30, 31]. Our nomogram also demonstrates that radiotherapy alone, or preoperative or postoperative radiotherapies, could improve the OS rate of patients with KTSCC, but radiotherapy before and after surgery was a risk factor for imaging prognosis, indicating that the sequence and status of radiotherapy also have greater impacts on KTSCC prognosis. This may be related to excessive radiotherapy, which can destroy local normal tissues, cause swallowing disorders, hearing disorders, and other discomforts, or even accelerate tumor metastasis. Clinical treatments should therefore integrate various factors, control the frequency and sequence of radiotherapy within an appropriate range, and seek a trade-off between prolonging survival time and maintaining the quality of life. This will also be addressed in the next step of our research, that is, designing prospective experiments to clarify the relationship between radiotherapy and KTSCC prognosis.

We have established a new predictive model (Figure 2) that was mostly based on individual patient conditions to predict the probabilities of OS at 3-, 5-, and 8- year after the diagnosis of KTSCC in individual patients. Clinical medical staff can calculate the total score for the nomogram based on the combined situation of each patient and then make more reasonable and reliable decisions based on the survival probability corresponding to that total score.

C-index and AUC were used to test the discrimination of the new model, which would be found to have better discrimination ability. The results for the quantitative indicators NRI and IDI demonstrated that the accuracy of our model in predicting survival probabilities was significantly better than that of the AJCC staging system. The calibration curve plot of this model was found to be highly consistent with the standard curve, and the DCA plot indicated that the net benefit of our model in predicting the prognosis of patients with KTSCC was superior to that of the AJCC staging system [32]. This suggests that our model is of greater value in clinical applications.

This study had certain shortcomings. Selection bias: this study is a retrospective design, which can easily lead to selection and information biases. More sociological and biological factors need to be included in the research: clinicopathological factors, certain biological indicators, and patient behavior habits that affect prognosis are not included in the SEER database. Further confirmation is needed in prospective studies: prospective studies of the developed nomogram are still needed to further test and confirm its predictive power, which will be the focus of our future research.

## 5. Conclusion

This study has successfully established and verified a prognostic nomogram for OS in KTSCC. Notably, age and the sequence and status of radiotherapy significantly affected KTSCC prognosis. The nomogram can predict the 3-, 5-, and 8-year os of KTSCC patients. Therefore, it can help clinicians to formulate more reasonable treatment strategies.

## Figures and Tables

**Table 1:** Lists the basic information for the two cohorts.

Variable	Training cohort	Validation cohort	*p*-value
Number of patients *n* (%)	2711 (70)	1163 (30)	
Age of diagnosis	61 (52–71)	61 (53–71)	**0.860**
Sex *n* (%)			**0.323**
Male	1696 (62.6)	747 (64.2)	
Female	1015 (37.4)	416 (35.7)	
Race *n* (%)			**0.146**
White	2292 (84.6)	1003 (86.2)	
Black	166 (6.1)	74 (6.3)	
Other	253 (9.3)	86 (7.3)	
Marital status *n* (%)			**0.371**
Married	1535 (56.6)	686 (58.9)	
Single	571 (21.0)	227 (19.6)	
Other	605 (22.4)	250 (21.5)	
Size *n* (%)			**0.456**
≤2	1102 (40.6)	471 (40.5)	
[2, 4]	871 (32.2)	355 (30.5)	
≥4	738 (27.2)	337 (28.9)	
AJCC stage *n* (%)			**0.971**
I	842 (31.1)	369 (31.7)	
II	458 (16.9)	194 (16.6)	
III	522 (19.3)	224 (19.3)	
IV	889 (32.7)	376 (32.4)	
Combined summary stage *n* (%)			**0.229**
Localized	1247 (45.9)	558 (47.9)	
Regional	1093 (40.4)	435 (37.4)	
Distant	371 (13.7)	170 (14.7)	
Surgery *n* (%)			**0.706**
Yes	2156 (79.6)	911 (78.4)	
No/unknown	555 (20.4)	252 (21.6)	
Radiotherapy *n* (%)			**0.541**
Yes	1416 (52.3)	595 (51.2)	
No/unknown	1295 (47.7)	568 (48.8)	
Chemotherapy *n* (%)			**0.084**
Yes	852 (31.5)	371 (31.9)	
No/unknown	1859 (68.5)	792 (68.1)	
RX summ—scope reg LNSur *n* (%)			**0.351**
Yes	1559 (57.5)	650 (55.8)	
No/unknown	1152 (42.5)	513 (44.2)	
RX summ—surg/rad seq *n* (%)			**0.783**
No radiation	1707 (62.9)	742 (63.8)	
Radiation prior to surgery	27 (0.9)	10 (0.8)	
Radiation after surgery	956 (35.4)	405 (34.8)	
Radiation before and after surgery	21 (0.8)	6 (0.6)	
Income *n* (%)			**0.537**
<$55,000	496 (18.3)	216 (18.5)	
[$55,000, $75,000]	1386 (51.2)	573 (49.3)	
>$75,000	829 (30.5)	374 (32.2)	

*Note.* RX summ—scope reg LN sur: regional lymph node dissection after surgery; RX summ—surg/rad seq: surgery and radiation sequence.

**Table 2:** Selected variables by multivariable cox regression analysis.

Variable	Multivariable analysis		
	HR	95% CI	*p*-value
Age of diagnosis	1.032	[1.026, 1.036]	**p** **≤** **0.001**^*∗∗∗*^
Sex			
Male	Reference		
Female	0.886	[0.793, 0.990]	**0.033** ^*∗*^
Race			
White	Reference		
Black	1.238	[1.021, 1.501]	**0.030** ^*∗*^
Other	1.144	[0.953, 1.372]	**0.148**
Marital status			
Married	Reference		
Single	1.553	[1.357, 1.777]	**p** **≤** **0.001**^*∗∗∗*^
Other	1.353	[1.194, 1.534]	**p** **≤** **0.001**^*∗∗∗*^
Size			
≤2	Reference		
[2, 4]	1.346	[1.118, 1.620]	**p** **≤** **0.001**^*∗∗*^
≥4	1.768	[1.486, 2.104]	**p** **≤** **0.001**^*∗∗∗*^
AJCC stage			
I	Reference		
II	1.348	[1.058, 1.718]	**0.015** ^*∗*^
III	1.379	[1.043, 1.823]	**0.023** ^*∗*^
IV	1.586	[1.171, 2.148]	**0.003** ^*∗∗*^
Combined summary stage			
Localized	Reference		
Regional	1.342	[1.082, 1.666]	**0.007** ^*∗∗*^
Distant	1.951	[1.501, 2.536]	**p** **≤** **0.001**^*∗∗∗*^
Surgery			
Yes	Reference		
No/unknown	1.150	[0.930, 1.422]	**0.195**
Radiotherapy			
Yes	Reference		
No/unknown	1.543	[1.240, 1.921]	**p** **≤** **0.001**^*∗∗∗*^
Chemotherapy			
Yes	Reference		
No/unknown	1.111	[0.958, 1.288]	**0.162**
RX summ—scope reg LN sur			
Yes	Reference		
No/unknown	1.196	[1.043, 1.371]	**0.010** ^*∗*^
RX summ—surg/rad seq			
No radiation	Reference		
Radiation prior to surgery	0.988	[0.605, 1.615]	**0.964**
Radiation after surgery	1.185	[0.957, 1.468]	**0.117**
Radiation before and after surgery	2.429	[1.468, 4.019]	**p** **≤** **0.001**^*∗∗∗*^
Income			
<$55,000	Reference		
[$55,000, $75,000]	1.020	[0.890, 1.169]	**0.773**
>$75,000	0.945	[0.813, 1.100]	**0.470**

*Notes*. ^*∗*^*p* < 0.05; ^*∗∗*^*p* < 0.01; ^*∗∗∗*^*p* ≤ 0.001, HR: hazard ratio, CI: confidence interval, RX summ—scope reg LN sur: regional lymph node dissection after surgery; RX summ—surg/rad seq: surgery and radiation sequence.

## Data Availability

The datasets generated and analyzed during the current study are available in the SEER database (https://seer.cancer.gov/). SEER is supported by the Surveillance Research Program (SRP) of the NCI Division of Cancer Control and Population Sciences (DCCPS). The aim is to inform the science of cancer surveillance and the collection, analysis, interpretation, and dissemination of reliable population-based statistics. SEER releases a standard set of research data every spring based on the previous November's submission of data from the registries. The data we used is based on the November 2021 submission. We accessed these through the SEER*∗*Stat software with additional approvals. The data that support the findings of this study are available from the SEER*∗*Stat software, but restrictions apply to the availability of these data, which were used under license for the current study, and so are not publicly available. Data are, however, available upon reasonable request and with the SEER Research Data Agreement.

## Abbreviations

KTSCC: Keratinizing tongue squamous cell carcinoma
TSCC: Tongue squamous cell carcinoma
SEER: Surveillance, epidemiology, and end results
C-index: The concordance index
AUC: Area under the time-dependent receiver operating characteristic curve
NRI: Net reclassification index
IDI: Integrated discrimination improvement
DCA: Decision-curve analysis
AJCC: American joint committee on cancer
OS: Overall survival
HR: Hazard ratio
CI: Confidence interval.
